# Alopecia Areata Is Associated with an Increased Risk for Prediabetes and Obesity: A Nationwide Case—Control Study

**DOI:** 10.3390/jpm15010016

**Published:** 2025-01-03

**Authors:** Yonit Wohl, Jacob Mashiah, Oberkovich Noy, Yarden Drutin, Shiraz Vered, Amir Ben-Tov

**Affiliations:** 1 Faculty of Medicine, Ariel University, Ariel 4070000, Israel; nnoyalle@gmail.com; 2 Maccabi Health Services, Tel-Aviv 6812509, Israel; 3 Kahn Sagol Maccabi Research and Innovation Center, Maccabi Healthcare Services, Tel-Aviv 6812509, Israel; 4 Pediatric Dermatology Unit, Division of Dermatology, Tel Aviv Sourasky Medical Center, Tel Aviv 6423906, Israel; 5 Sackler Faculty of Medicine, Tel Aviv University, Tel Aviv 6997801, Israel; 6 Department of Dermatology and Reference Center for Genodermatoses and Rare Skin Diseases (MAGEC), Necker-Enfants Malades Hospital, 75015 Paris, France; 7 Pediatrics Department, Dana-Dwek Children’s Hospital, Tel Aviv Sourasky Medical Center, Tel Aviv 6423906, Israel; 8 School of Public Health, University of Haifa, Haifa 3103301, Israel

**Keywords:** alopecia areata, prediabetes, obesity, increased risk

## Abstract

**Background and Aims:** Alopecia areata (AA) is a non-scarring inflammatory hair loss condition associated with various immune-mediated comorbidities. Prediabetes, characterized by elevated blood glucose levels not yet high enough to be classified as diabetes, significantly increases the risk of developing type 2 diabetes mellitus (T2DM) and cardiovascular complications. The associations between AA obesity and prediabetes have long been investigated in an attempt to identify preventable risk factors, yet the literature is relatively scarce and inconclusive. This study aimed to explore the association between AA, prediabetes, obesity, and T2DM in a large population cohort. **Methods:** All patients diagnosed with AA between 2005 and 2019 within Maccabi Healthcare Services (MHS) in Israel were compared with age-matched and gender-matched healthy controls for prediabetes, T2DM and obesity, using logistic regression models for all analyses. A total of 33,401 patients with AA and 66,802 controls were included in the analysis. **Results:** The prevalence of prediabetes was significantly higher in AA patients (26.3%) compared to controls (18.1%), with an odds ratio (OR) of 1.62. Obesity prevalence was also higher in AA patients (17.2% vs. 13.3%, OR 1.35). T2DM prevalence was similar between groups. Prediabetes prevalence notably increased with age in AA patients, especially in those aged 40 and older (OR 2.02). **Discussion:** The study highlights a significant association between prediabetes and alopecia areata, with prediabetes risk emerging prominently in AA patients. Obesity also showed a strong link with AA. These findings suggest the need for regular screening and early management of prediabetes and obesity in patients with AA to potentially mitigate associated health risks.

## 1. Background

Alopecia areata (AA) is a common, non-scarring inflammatory alopecia which is associated with a growing number of immune-mediated comorbidities and has complex immunopathogenic etiology. The onset of the disease ranges but is most prevalent in the second and third decades. Current assessments of the prevalence of alopecia areata in adults range from 0.2% to 2%, increasing over the years and affecting both sexes equally [1].

Genetics and environmental factors interplay through different immune pathways and inflammatory cytokines (e.g., interferon-γ and interleukin 15) that signal through Janus kinases, thus may lead to AA and other autoimmune diseases [2].

A growing number of systemic and cutaneous immune mediated comorbidities are reported to be associated with AA, such as but not limited to systemic lupus erythematosus, vitiligo, Sjogren disease, psoriasis, atopic dermatitis, inflammatory bowel disease, and rheumatoid arthritis [3].

According to the updated literature, overall, patients with AA have three times higher odds ratios of developing any autoimmune/inflammatory disease than patients without AA [3].

Lately, a few modifiable metabolic disorders such as prediabetes, obesity, and DM, with a major impact on one’s health are suspected to be potential risk factors for AA [4].

Prediabetes is a condition characterized by blood glucose levels that are higher than normal, but not yet high enough to be classified as diabetes. It is an intermediate metabolic state that significantly increases the risk of developing type 2 diabetes mellitus (T2DM) and cardiovascular complications [5]. Early identification and management of prediabetes, which is often asymptomatic, is crucial to prevent or delay the progression to diabetes and associated health consequences [6].

Obesity, defined by a body mass index (BMI) over 30, is a chronic multifactorial disease characterized by excessive accumulation of body fat, which poses significant risks to health.

Rates of overweight and obesity are rising globally in adults and children. From 1990 to 2022, the percentage of children and adolescents aged 5–19 years living with obesity increased four-fold, from 2% to 8% globally, while the percentage of adults 18 years of age and older living with obesity more than doubled from 7% to 16%.

Overweight and obesity are major risk factors for cardiovascular diseases which are the leading causes of death worldwide. Diabetes and its associated conditions, such as musculoskeletal disorders including osteoarthritis, and the risk for development certain solid cancers are also increased [7].

Prediabetes and obesity constitute significant components of the metabolic syndrome (MS), and together and alone, represent major public health challenges globally due to their escalating prevalence, association with multiple comorbidities, and cardiovascular sequela.

Low-grade chronic inflammation is at the root of metabolic syndrome immunopathogenesis. In short, a variety of proinflammatory cytokines are secreted by adipocytes in obese individuals, generating insulin resistance and defective insulin secretion, which further contribute to T2DM and metabolic syndrome, leading to maladaptive responses such as fibrosis and necrosis that can cause significant tissue damage [8].

Considering the common inflammatory pathogenesis, and in order to identify and prevent risk factors for the above metabolic disorders, the associations between AA obesity and prediabetes have long been investigated, yet the literature is relatively scarce and inconclusive.

T2DM has been reported to be more prevalent in patients with AA, in previous series of epidemiologic AA cohort studies including different populations [9,10].

Data on the prevalence of metabolic syndrome in AA patients has been more conflicting. While some comparative studies showed patients with AA are more likely to exhibit multiple components of metabolic syndrome, such as abdominal obesity, elevated blood pressure, dyslipidemia, and impaired glucose metabolism [11,12], other studies found similar prevalence of MS among cases of AA and healthy controls or did not find any effect of BMI on the disease course of AA [13,14].

### Aims

We aimed to explore and elucidate the association between alopecia areata and prediabetes, obesity, and T2DM in a large population cohort.

## 2. Methods

We conducted a population-based retrospective case–control cohort study that included all patients diagnosed with alopecia areata between 2005 and 2019 in Maccabi Healthcare Services (MHS), a large health fund in Israel, covering roughly 26% of the nation’s population. Patients with alopecia areata were identified, matched in a 1:3 ratio, and compared with healthy controls. Matching was performed using the following: gender, age, and geographical area (MHS branch), which closely reflects socioeconomic status. A total of 33,401 patients with alopecia areata and 66,802 controls were included in the analysis.

AA patients were identified using a clinical diagnosis code established by a board-certified dermatologist.

Prediabetes, T2DM, and Obesity were defined according to MHS registries. Patients with prediabetes were defined as patients with HbA1C above 5.7 or Oral glucose tolerance test with 75 g of glucose for 2 h with levels above 140 or two fasting glucose measurements above 100 mg/dl.

T2DM was defined as HbAlC > 6.5% or Glucose > 125 mg/dL or a diagnosis of diabetes (ICD9 code) in the chart. Obesity was defined as a body mass index above 30.

### 2.1. Statistical Analysis

Data were analyzed using SPSS version 29, and *p*-values < 0.05 were considered significant. Continuous variables were reported as mean ± SD and between-group comparisons were performed using a *t*-test for independent samples. Categorical data were reported as frequency, and percentage and associations were tested using Chi-square test. We performed a logistic regression analysis on prediabetes, T2DM and obesity to assess the odds ratios (OR) and 95% confidence intervals (CI) for the associations. Since matching was previously conducted using the aforementioned method, there was no need for further controlling.

### 2.2. Ethical Approval

The study protocol was approved by the Maccabi Healthcare Services institutional review board, ID number 0131-20 (date of approval 08/01/2020).

## 3. Results

Demographic characteristics are detailed in Table 1.

The average age at onset was similar between the groups (29.9 ± 16.9 years), as was the proportion of males (56.5%) and females (43.5%).

Socioeconomic status was defined according to the Israeli Central Bureau of Statistics (scale 1–10), combining geographic and socioeconomic information for each neighborhood in Israel.

Compared to controls, the proportion of Israeli-born people from the middle classes was higher in AA patients (87% vs. 80.6 and 56.1% vs. 54.6%, respectively, *p* < 0.001

Out of the total cohort, 20,863 {20.8%) patients had prediabetes, 4193 (4.2%) patients had T2DM, and 14,633 (14.6%) patients suffered from obesity (Table 2).

In total, the prevalence of prediabetes was significantly increased among patients with alopecia areata compared to the control patients, followed by an increased prevalence of obesity. T2DM was only slightly increased in AA patients compared to controls.

In a subgroup analysis by age group, differences remained significant in all age groups when either comorbidity was compared to controls,

Compared to controls, AA patients aged 40 and older exhibited a sharp increase in the prevalence of prediabetes: to more than double (55.4% vs. 38.2% (OR 2.02, CI (1.92–2.12), followed by a modest increase in prevalence of obesity (28% vs. 22%, odds ratio (OR) 1.38, 95% Confidence Interval (CI) 1.31–1.46), and no increase in T2DM prevalence (12.7% vs. 10.5%, odds ratio (OR) 1.23, 95% Confidence Interval (CI) 114–1.33), respectively. Other age groups exhibited modest elevated rates of prediabetes and obesity, except for T2DM in age < 18 where no difference was observed (Table 2).

Further analysis of the relative odds ratios of the studied variables and their various combinations among patients and controls are presented in Table 3.

## 4. Discussion

Alopecia areata is an inflammatory skin disease which affects nearly 2% of the general population at some point during their lifetime and is associated with various concomitant inflammatory disorders and environmental stimuli. Daily lifestyle factors such as smoking, excess body weight, alcohol consumption, diverse diet components, etc., may be involved in the onset and the worsening of inflammatory diseases. However, only a limited number of studies have investigated these influences on alopecia areata [15].

In the present study, we focused on a few modifiable metabolic disorders that are suspected to be potential risk factors for AA.

The association between AA and prediabetes has been investigated for a long time but is far from being determined.

However, in recent years, the link between the disorders has been established, as epidemiological evidence has accumulated through epidemiologic data and small case–control studies.

Karadag et al. were the first to report increased insulin resistance in AA patients based on measured fasting blood glucose (FBS), C-peptide, plasma insulin, and homeostasis model assessment for insulin resistance (HOMA-IR) in 60 AA patients and controls [9].

Similar results were reproduced in another study by Shadidi et al. who found that plasma levels of insulin, C-peptide, and HOMA-IR were significantly higher in patients with AA compared to controls and patients with a more severe disease, i.e., alopecia totalis/universalis, had higher levels of insulin compared to those with patchy hair loss [16].

Lately, research has focused on different metabolic components such as serum levels of lipocalin, insulin, adiponectin, and leptin, which were examined and found to be higher in subgroups of AA patients, thus may serve as biomarkers for AA and correlate with disease severity [17,18,19].

In this study, we support previous data by finding significantly higher rates of prediabetes and obesity among AA patients compared with controls in a very large representative cohort. To elucidate the contribution of each comorbidity to the elevated risk for AA, we clearly show that prediabetes is the dominant risk factor.

Although not proving causality, this strong association may suggest common inflammatory responses and mediators.

Also, the augmented risk late in adulthood may further imply on several immune mechanisms of inflammation such as autoreactive pathways, epitope spreading and other, accountable of the concurrent display.

Interestingly, analyzing different combinations of the risk factors in our study which does not show augmented risk relative to their associations when studied alone, may suggest that they do not work by synergistic mechanisms in AA patients.

The precise pathophysiology of AA is complex and not fully understood. However, it is widely accepted that AA results from an aberrant immune response targeting hair follicles. Cytotoxic T lymphocytes infiltrate hair follicles and initiate the hair loss process.

Different immune cell lines including plasmacytoid dendritic cells, natural killer cells and T cells, along with key molecules such as interferon-γ, interleukin-15, MICA, and NKG2D, transforming growth factor beta/Tregs and JAK kinase signaling, have been identified as contributing to the complex autoimmune process [20,21].

Type 2 diabetes is characterized by an ongoing cytokine-mediated acute phase response initiated by the innate immune system, and continues with the adaptive immune system.

Inflammatory regulation has focused on innate immunity especially macrophage for a long time; while increasing evidence suggests that T cells are crucial for the development of metabolic inflammation and insulin resistance, including all arms of TH lymphocytes and regulatory T cells [22].

Furthermore, varying dynamics of the immune system seems to occur with the advancing disease, enabling differentiation of the early preclinical and clinical phases of the disease (i.e., prediabetes), diabetes complications, and disease progression [23].

In particular, elevated circulating inflammatory markers such as C-reactive protein (CRP) and interleukin-6 (IL-6) predict the development of type 2 diabetes mellitus [24]. Also, the process of aging itself is associated with immunological changes, immune senescence, accompanied by a chronic inflammatory state which may contribute to metabolic syndromes, diabetes, and their cardiovascular consequences.

Our study results also demonstrate AA is positively associated with obesity, supporting earlier research on this link. Although Yosipovitz et al. demonstrated that the skin barrier function is impaired in obesity [25], clinical data and experimental models have focused on the immune system, specifically in the involvement of adipokines in the pathogenesis of various autoimmune diseases, suggesting that obesity may be a major environmental factor contributing to the onset and progression of autoimmune diseases, including alopecia areata.

IL-17 is believed to be the dominant mechanism of obesity-related cutaneous inflammation, although, Th2-mediated immune response may possibly relate to the pathogenesis of alopecia areata as well [26]. Nevertheless, recent research continues to investigate the possible link between alopecia areata and adipokines, especially adiponectin and leptin [27].

Strengths of our study include its population-based setting, a vast sample size, with demographic diverse representation and validated dermatologic case definition of most AA cases.

The limitations of the present study include a lack of analysis by subgroups of disease severity, and although representative of population diversity, this is a single country-based epidemiologic study. Also, family history; temporal occurrence of concurrent inflammatory disorders; lifestyle factors such as smoking, dietary intake, physical activity and relevant medical treatment for each patient were not reported. Finally, our study design is retrospective and fails to establish causality

In this comparative study, we identified that AA patients have a significant risk for concomitant prediabetes, followed by a lesser degree risk for obesity.

Recently, treatment options for severe AA have expanded dramatically with the introduction of biologically targeted small molecule agents from the Jak inhibitor class to the pharmaceutical arsenal [28].

Despite this, considering our findings and that prediabetes and obesity are preventable disorders and highly associated with health consequences, we believe that efforts should be primarily focused on their early detection in all AA patients. Further management strategies should include lifestyle modifications, pharmacotherapy, and regular monitoring of blood glucose levels, HbA1c, blood pressure, BMI, lipid profiles, as well as screening for complications.

In summary, our data indicate that prediabetes and obesity are highly associated with alopecia areata, suggesting screening and monitoring efforts should be implemented in all AA patients in all age groups.

Continuous research, such as meta-analyses on different populations, is important and necessary to further evaluate the bidirectional associations between AA and different components of the metabolic syndrome.

Finally, long term studies are needed to determine whether earlier and/or more aggressive treatment of AA may limit the development of comorbidities, and to establish the most cost-effective treatment.

## Figures and Tables

**Table 1:** Demographic characteristics of Alopecia Areata patients and controls.

		All N = 100,203		Control N = 66,802		Case N = 33,401		*p* Value
		N	%	N	%	N	%	
Age diagnosis (mean ± std)		29.9 ± 16.9		29.9 ± 16.9		29.9 ± 16.9		1.000
Sex	Female	43,638	43.5	29,092	43.5	14,546	43.5	1.000
	Male	56,565	56.5	37,710	56.5	18,855	56.5	
Country of birth	Israel	82,873	82.7	53,829	80.6	29,044	87.0	<0.001
	Other	17,330	17.3	12,973	19.4	4357	13.0	
Socioeconomic status	Low (1–4)	20,711	20.7	13,517	20.2	7194	21.5	<0.001
	Med (5–7)	55,184	55.1	36,448	54.6	18,736	56.1	
	High (8–10)	24,308	24.3	16,837	25.2	7471	22.4	

**Table 2:** Odds ratios of Prediabetes. T2DM, Obesity, and Alopecia Areata patients compared to controls in different age groups.

Age at Alopecia Areata Diagnosis	Comorbidities	All N = 100,203		Control N = 66,802		Case N = 33,401		OR (95%CI)	*p*-Value
		N	%	N	%	N	%		
All (N = 100,203)	Prediabetes (vs. no prediabetes)	20,863	20.8	12,064	18.1	8799	26.3	1.62 [1.57–1.67]	<0.001
	Diabetes (vs. no diabetes)	4193	4.2	2613	3.9	1580	4.7	1.22 [1.14–1.30]	<0.001
	Obesity (vs. no obesity)	14,633	14.6	8903	13.3	5730	17.2	1.35 [1.30–1.39]	<0.001
Age < 18 (N = 27,363)	Prediabetes (vs. no prediabetes)	978	3.6	571	3.1	407	4.5	1.45 [1.27–1.65]	<0.001
	Diabetes (vs. no diabetes)	70	0.3	41	0.2	29	0.3	1.42 [0.89–2.28]	0.152
	Obesity (vs. no obesity)	953	3.5	591	3.2	362	4.0	1.23 [1.08–1.41]	0.002
Age 18–40 (N = 44,169)	Prediabetes (vs. no prediabetes)	7293	16.5	4198	14.3	3095	21.0	1.60 [1.52–1.69]	<0.001
	Diabetes (vs. no diabetes)	898	2.0	556	1.9	342	2.3	1.24 [1.08–1.42]	0.002
	Obesity (vs. no obesity)	6804	15.4	4114	14.0	2690	18.3	1.38 [1.31–1.45]	<0.001
Age 40+ (N = 28,671)	Prediabetes (vs. no prediabetes)	12,592	43.9	7295	38.2	5297	55.4	2.02 [1.92–2.12]	<0.001
	Diabetes (vs. no diabetes)	3225	11.2	2016	10.5	1209	12.7	1.23 [1.14–1.33]	<0.001
	Obesity (vs. no obesity)	6876	24.0	4198	22.0	2678	28.0	1.38 [1.31–1.46]	<0.001

**Table 3:** Odds ratios of Prediabetes. T2DM, Obesity, and various combinations among Alopecia Areata patients compared to controls.

	Comorbidities	All N = 100,203		Control N = 66,802		AA N = 33,401		OR (95%CI)	*p*-Value
		N	%	N	%	N	%		
All (N = 100,203)	No prediabetes, no T2DM, and no obesity	71,724	71.6	49,968	74.8	21,756	65.1	1.00 (Ref.)	-
	Only prediabetes	12,128	12.1	6879	10.3	5249	15.7	1.75 [1.69–1.82]	<0.001
	Only T2DM	690	0.7	463	0.7	227	0.7	1.13 [0.96–1.32]	0.145
	Only obesity	6242	6.2	3841	5.7	2401	7.2	1.44 [1.36–1.51]	<0.001
	Prediabetes and obesity	5916	5.9	3501	5.2	2415	7.2	1.58 [1.50–1.67]	<0.001
	T2DM and obesity	684	0.7	466	0.7	218	0.7	1.07 [0.91–1.26]	0.384

Again, prediabetes displays the strongest associations with AA, relative to obesity (OR 1.75 vs. OR 1.44), respectively. As expected, only when prediabetes and obesity are combined, an increased risk of AA is seen, relatively like that of prediabetes alone (OR 1.75 vs. OR 1.58).

## Data Availability

Data are unavailable due to privacy or ethical restrictions.
